# Macaques primed with self-amplifying RNA vaccines expressing HIV-1 envelope and boosted with recombinant protein show potent T- and B-cell responses

**DOI:** 10.1186/1742-4690-9-S2-P24

**Published:** 2012-09-13

**Authors:** WM Bogers, H Oostermeijer, P Mooij, G Koopman, E Verschoor, D Davis, JL Heeney, Y Cu, K Banerjee, B Burke, A Dey, A Geall, SW Barnett

**Affiliations:** 1 Biomedical Primate Research Centre, Rijswijk, the Netherlands; 2 University of Cambridge, Cambridge, UK; 3 Novartis Vacccines and Diagnostics, Inc., Cambridge, USA

## Background

Self-amplifying RNAs (replicons) of positive-strand viruses are useful vectors for delivering vaccine antigens. Novartis has developed a self-amplifying mRNA (SAM™) vaccine platform to take advantage of cell-free RNA production and synthetic non-viral delivery systems. In this study, the safety, immunogenicity, and efficacy of HIV-SAM™ vaccines encoding HIV-1 clade C TV1 gp140 envelope glycoprotein were evaluated in rhesus macaques using two non-viral delivery systems: lipid nanoparticle (LNP) and a Novartis proprietary 2nd generation delivery technology (CNE).

## Methods

Five groups of six macaques were primed at weeks 0, 4 and 12 with HIV-SAM™ vaccine formulated with LNP or CNE, alphavirus replicon particles (VRP), recombinant TV1 gp140 glycoprotein in MF59 adjuvant, or with vector controls encoding an irrelevant Ag. All treatment groups were boosted intra-muscularly at weeks 24 and 36 with TV1 gp140 in MF59, and controls with irrelevant protein in the same adjuvant. Systemic and mucosal responses were measured throughout the study. All macaques will be given a repeated low dose intra-rectal challenge with the heterologous clade C SHIV-1157ipd3N4 challenge.

## Results

After priming immunizations, both IFNγ and IL2 T-cell responses and B-cell ELISpots were higher in HIV-SAM™-CNE macaques than those in HIV-SAM™-LNP, VRP and glycoprotein alone groups. Systemic Env-specific antibody responses were also detected by ELISA at week 6 in the RNA-immunized groups and increased after subsequent immunizations. Neutralization, ADCC, epitope mapping, and antibody isotyping assays are underway to further evaluate the antibody responses in these animals. No adverse responses to RNA immunizations were observed.

## Conclusion

These studies provide the first evidence in nonhuman primates that vaccination with formulated self-amplifying RNA is safe and immunogenic, eliciting both humoral and cellular immune responses.

This study was supported by NIH grant 5 PO1 AI066287-02.

